# Axonal transport deficits in multiple sclerosis: spiraling into the abyss

**DOI:** 10.1007/s00401-017-1697-7

**Published:** 2017-03-18

**Authors:** Robert van den Berg, Casper C. Hoogenraad, Rogier Q. Hintzen

**Affiliations:** 1 Department Of Neurology, Erasmus MC Rotterdam, Rotterdam, The Netherlands; 2 Cell Biology, Utrecht University, Utrecht, The Netherlands

**Keywords:** Axonal transport, Kinesin, Dynein, Mitochondrial transport, Neurodegeneration, Microtubule, Intracellular transport

## Abstract

The transport of mitochondria and other cellular components along the axonal microtubule cytoskeleton plays an essential role in neuronal survival. Defects in this system have been linked to a large number of neurological disorders. In multiple sclerosis (MS) and associated models such as experimental autoimmune encephalomyelitis (EAE), alterations in axonal transport have been shown to exist before neurodegeneration occurs. Genome-wide association (GWA) studies have linked several motor proteins to MS susceptibility, while neuropathological studies have shown accumulations of proteins and organelles suggestive for transport deficits. A reduced effectiveness of axonal transport can lead to neurodegeneration through inhibition of mitochondrial motility, disruption of axoglial interaction or prevention of remyelination. In MS, demyelination leads to dysregulation of axonal transport, aggravated by the effects of TNF-alpha, nitric oxide and glutamate on the cytoskeleton. The combined effect of all these pathways is a vicious cycle in which a defective axonal transport system leads to an increase in ATP consumption through loss of membrane organization and a reduction in available ATP through inhibition of mitochondrial transport, resulting in even further inhibition of transport. The persistent activity of this positive feedback loop contributes to neurodegeneration in MS.

## Introduction

To develop new therapeutic approaches towards curing any given disease, it is necessary to gain an understanding of the mechanisms that cause the illness, contribute to its progression and prevent its treatment. For multiple sclerosis (MS), an incurable neurological disorder mainly affecting young adults, medical science is still struggling to grasp the underlying pathological processes. The inflammatory component of the disease is obvious, and this is further supported by the recently identified risk genes that play a role in both adaptive and innate immunity [89, 107]. Though the neurodegenerative component of the disease had already been described in the 19th century—even in the original descriptions by Charcot [24]—there is a recent revival of research interest in the CNS tissue component of the disease. Several observations suggested that neurodegeneration plays a central role in disease development and progression. EM studies have shown that myelin degeneration starts at the inner myelin sheaths, instead of on the outside as would be expected if caused by an external immune response [123]. Also, there are strong indications that disturbed axoglial support can feed neurodegenerative processes [144]. Already at the time of the first attack in children, axoglial proteins appear abundant in CSF [134]. As shown in non-human primates, such markers of CNS tissue damage can induce further autoimmune neurological disease [55].

Furthermore, there are indications that once neurodegenerative damage occurs, this progresses in an autonomous mode, irrespective of adaptive immune reactions [43]. This may account for the observation that immune modulating drugs, though quite effective in suppressing inflammatory attacks, fail to halt progression, especially when started in later phases when demyelinating damage has already occurred [56]. A better understanding of the mechanisms involved in the neurodegenerative component of MS could potentially lead to new treatments, aimed at slowing down or preventing the disability caused by neuronal loss.

The cellular processes enabling neurons to generate and transmit signals demand high amounts of energy in the form of ATP. At the same time the central nervous system contains very little energy reserves [4]. This combination makes neurons highly vulnerable to energy deficits, which are considered to play a crucial role in neurodegeneration. As in other cell types, the main energy source of the neuron is ATP production by the mitochondria, with defects in mitochondrial function being highly associated with neurodegeneration (reviewed in [90]). During the course of MS, neurons become even more vulnerable to energy deficits when they lose their myelin sheaths, increasing the energy needed to propagate a signal along the axon and to maintain the action potential [19, 145].

Not only do neurons have a high energy demand, this demand is also distributed unevenly throughout the cell and shifts over time, depending on the activity of the cell and its neighbors. To facilitate the need for ATP, mitochondria are transported by motor proteins along the cytoskeleton to areas with high cellular activity, where they are anchored to the microtubule [13, 18, 129, 149]. Defects in this transport system are found in a large variety of neurodegenerative disorders including Alzheimer’s disease [122], amyotrophic lateral sclerosis (ALS, [11]) and Huntington’s disease [87], and are now recognized to be one of the main underlying mechanisms of neurodegeneration [31, 103, 106].

For the transport system to function with high efficiency required to maintain neuronal integrity, at least three conditions should be met. First of all, the infrastructure along which transport takes place, consisting of microtubules [152] and actin networks [25], must be intact for a cargo to reach its destination. However, infrastructure is useless without transporters to use it. Therefore, the motors and their adaptor proteins must be present in sufficient numbers and fully functional [59, 127]. Finally, for any complex transport system to work efficiently, control and guidance mechanisms should be in place [99, 150]. For axonal transport, regulation takes place through various mechanisms including chemical modification of the microtubules [52, 68], calcium sensor proteins [18, 98] and anchoring proteins docking cargo at the desired destination [17, 73]. Defects in any part of this system reduce its effectiveness and put the neuron at risk for degeneration.

A role for axonal transport in neurodegeneration has been shown to exist in a large number of different neurological disorders. In this review, we explore the current evidence pointing at axonal transport deficits in MS and discuss several mechanisms that can explain its role. Based on the increased understanding of these mechanisms, we will propose several scientific and therapeutic approaches which might be of interest to the MS research field in the coming decade.

## Axonal transport in multiple sclerosis

To understand the molecular basis of neuronal dysfunction in disease, much depends on the selection of a proper model. Experimental autoimmune encephalomyelitis (EAE) is a commonly used model for MS [8]. Many variants exists, with the common characteristic that through injection of a myelin component, combined with an immunogenic adjuvant, an immune response is triggered against the CNS myelin [48]. Although the resulting disease exhibits more characteristics of an inflammatory neuropathy than a true demyelinating disease of the central nervous system, it remains the closest animal model available for MS [7, 119]. Interestingly, deficits in axonal transport are one of the earliest pathological findings in EAE, preceding structural abnormalities and other signs of axonal degeneration. Even before demyelination took place, both anterograde and retrograde transport of mitochondria was drastically reduced and remained down-regulated for weeks in a chronic EAE model [136]. This is in line with earlier findings in the optical nerve of EAE animals, where a reduced transport of radioactive markers [51] and manganese ions [91] were shown. These EAE findings suggest that alterations in axonal transport form one of the first steps towards loss of the axon in multiple sclerosis models [32].

Many different factors contribute to MS susceptibility, including gender, environment, exposure of the immune system to pathogens and genetics (reviewed in [10, 27]). For decades, the only genetic risk factors known for MS were variations in the genes encoding human leukocyte antigens (HLAs, [69]). However, in recent years more than 110 genetic variants outside of the histocompatibility complexes have been found that each contribute to susceptibility to MS (reviewed in [126]). The vast majority of the proteins encoded by these genes either have a function in the immune system, primarily as part of a signaling pathway. Interestingly, several of the genes that do not fall in this category are involved in axonal transport. This includes three kinesin family member proteins (KIFs), the molecular motors responsible for anterograde transport along microtubules.

One of the KIFs that have been studied in the context of MS is Kif1b, a motor protein with at least two isoforms transporting mitochondria and synaptic vesicles [28, 104]. An effect of a SNP in Kif1b on MS susceptibility was first identified in a genetically isolated Dutch population. It was replicated in the same study in a second Dutch, a Swedish and a Canadian cohort [6]. Previous studies had already implicated this specific kinesin in the pathogenesis of Charcot-Marie-Tooth disease type 2A (CMT2A, [161]), a disorder of the peripheral nervous system showing myelin degeneration and associated with axonal transport impairments [44], suggesting that this mechanism was shared by both disorders. A more recent study showed that in zebrafish, Kif1b is essential for development of myelinated axons [96]. However, several attempts to replicate the genetic association, including a large multicenter study [16], failed to show a similar effect. Although this difference could be a consequence of the specific characteristics of this Dutch population, in which the control allele frequency of the gene was significantly lower than in the general population [58], it does appear that this gene does not contribute to MS susceptibility worldwide [50].

A second KIF was implicated when a large international consortium found an association between Kif21b and MS susceptibility [66], later replicated in an independent Belgian cohort [49]. In neurons, Kif21b is enriched in the dendrites and involved in the transport of vesicles containing GABA receptor subunits [83]. It is similar in structure to Kif21a, a kinesin associated with congenital fibrosis of the extra-ocular muscles type 1 (CFEOM1, [157]). Interestingly, rather than performing a transport function, Kif21a acts as a regulator of the cytoskeleton, inhibiting microtubule growth at the cell cortex [148]. A recent study shows that kif21b can act as both a transporter and a microtubule regulator, depending on the level of neuronal activity [46]. Kif21b has also been found associated with an increased susceptibility to other autoimmune disorders such as rheumatoid arthritis [88].

A third association was found in a Spanish study, which identified a SNP in Kif5a as a risk factor for MS [3]. Kif5a is involved in the transport of neurofilament and mitochondria along microtubules [156] and forms a dimer with either Kif5b or Kif5c to transport mitochondria in axons [128]. In zebrafish, cooperation between Kif5a and Kif1b is essential for maintaining axon integrity, with Kif5a taking over part of the tasks of Kif1b when this protein is lost and vice versa [21]. Kif5a has been implicated in several axonopathies, including CMT2 [93]. These findings strongly suggest that variations in Kif5a can contribute to neurodegeneration in MS, especially in combination with similar variations in Kif1b and/or Kif21b.

### Transport deficits in MS neuropathology

A histopathological hallmark of a defective axonal transport system is the accumulation of organelles and proteins, resulting in detectable aggregates and axonal swelling. A commonly used marker for disrupted axonal transport is the amyloid precursor protein (APP). In healthy neurons, this protein is transported through the axon to its final location in the synapse [118]. If the transport system works at suboptimal efficiency, APP will accumulate in the axon. Several studies have reported that such accumulations can indeed be observed in post-mortem investigations of the brains of MS patients [41], in some cases independent of demyelination [12]. APP accumulation occurs already during the early phases of the disease, with the number of APP-positive axons showing a positive correlation with disease duration [82]. A detailed study using both APP and the synaptic vesicle protein SPY as markers for axonal transport, found that accumulation of these proteins occurs not only in active demyelinating lesions, but also in normal appearing white matter. Also, the motor protein KIF5A, as well as its associated cargo is found reduced in MS white matter [54], suggesting a reduced activity of the axonal transport system. Since the intracellular transport system is essential in developing and maintaining dendritic spines [147], a reduced efficiency of this system might also explain the recent observation that the number of spines is significantly reduced in the cortex of MS patients [71].

A special challenge for intracellular transport is to match the distribution of mitochondria to the local ATP consumption. Although mitochondria are essential for ATP production, aging mitochondria become a source of radical oxygen species (ROS) contributing to neurodegeneration [20, 40]. The axonal transport system is able to distinguish decaying mitochondria based on their membrane potential, moving active mitochondria with a high membrane potential to the sites where ATP is required. In contrast organelles with a low membrane potential will be transported back to the cell body for autophagy [40]. This means that both a reduced number of mitochondria as well as a persistence of aging mitochondria at the cell periphery pose a threat to axonal integrity. A number of post-mortem studies have shown that both situations can exist in the brains of MS patients. Several components of the mitochondrial respiratory chain were found reduced in activity, including complex I [94], III [34] and IV [101]. Demyelinated axons in the brains of MS patients were found to have a greater mitochondrial mass compared to myelinated axons and a higher expression of the docking protein syntaphilin [101]. This might initially be a protective mechanism. A larger number of mitochondria is transported to meet the higher ATP demand and is actively anchored to the microtubule network, prolonging the period the neuron can survive without myelin [111]. The mitochondrial density slightly decreases when axons are remyelinated, but remains high compared to myelinated axons. This higher density is entirely due to a larger number of stationary mitochondria, with the number of mobile mitochondria actually decreasing upon demyelination [159]. Taken together, these studies suggest that transport of mitochondria plays an important role in the neuronal response to the energy deficit faced in MS lesions.

## Transport deficits aggravate neurodegeneration

The previous paragraphs have shown that there is clear evidence for transport deficits in MS. It is not so clear, however, how these deficits lead to axonal loss. One straightforward explanation would be that a reduced transport of active mitochondria leads to reduced ATP production in the axon. In demyelinated axons of MS patients, sodium channels normally restricted to the nodes of Ranvier are now expressed along large regions of the axon [29]. A similar ectopic distribution is reported for calcium channels [80]. This will put additional strain on the neuron, since to maintain its membrane potential and prevent calcium toxicity both sodium and calcium have to be transported out of the cell using ATP-consuming transporters. Once ATP supplies are depleted, calcium in the axoplasm will rise to toxic levels, initiating a cascade resulting in axonal loss (reviewed in [138, 145]). Initially, the neuron will respond to the increased energy need by stimulating the transport of mitochondria, as observed in vitro [79] and in MS tissue [111], leading to an increased density of mitochondria in dysmyelinated axons of MS patients [19, 100, 154]. Although the cell is able to maintain its axon for a short period in this ‘overload mode’, if the situation persists for too long, local ATP supplies will fall and axon integrity will be lost. This hypothesis is supported by the observation that reduced mitochondrial mobility alone, without accompanying stress to the neuron, is sufficient to cause neurodegeneration [110].

As mentioned before, demyelinated axons show an increased expression of syntaphilin, a protein thought to anchor mitochondria to the microtubules, prohibiting their transport and providing a stable local source of ATP [101]. The *shiverer* mouse, which shows severe dysmyelination of the CNS, is considered a model for progressive MS due to the metabolic challenges its axons face as a result of chronic myelin loss. As in MS patients, a highly significant upregulation of syntaphilin was observed in axons of this mouse, associated with an increase in non-motile mitochondria. Interestingly, reducing the expression of syntaphilin by crossing the *shiverer* mouse with a syntaphilin knock-out line enhanced the transport of mitochondria from the axon back to the soma. Moreover, syntaphilin deletion also proved protective against both gray and white matter damage in the mouse, although it did not influence the outcome of EAE [70]. This indicates that a drug interfering with the binding between syntaphilin and either mitochondria or microtubule might theoretically reduce neurodegeneration in progressive multiple sclerosis by improving mitochondria mobility.

Mitochondria are just one of the cargoes transported along microtubules. Vesicles containing a large variety of proteins, mRNA and membrane lipids are ferried through the cell. In the oligodendrocyte, the transport of mRNA by kinesins along microtubules is essential for the proper production of myelin [9, 22, 96]. However, production alone is not enough to myelinate an axon. A complex interplay is required between oligodendrocyte and axon, communicating through cell–cell adhesion molecules (reviewed in [130, 133]). These adhesion molecules are organized in sharply demarcated membrane domains through interaction of the adapter protein 4.1B with the underlying actin cytoskeleton [37, 61]. Formation of these domains, the so-called paranodal junctions, is highly dependent on contactin-associated protein (Caspr [38]). Interestingly, the *shm* mouse in which the axonal transport of Caspr has been disrupted shows a distortion of myelin sheaths in the central nervous system, resulting in a reduced conduction velocity and a neurological disorder characterized by ataxia and hind limb paresis [141]. Downregulation of Caspr has also been observed in MS lesions, where it is considered an early sign of impending myelin loss [155]. This suggests a different route through which disruption of axonal transport can contribute to neurodegeneration, by cutting off the supply of adhesion molecules required to maintain the neuron-oligodendrocyte.

Even though an axon denuded of its myelin is at high risk of degeneration, if it is able to survive, remyelination might occur [42]. As is the case in the initial myelination of the axon, remyelination depends on a number of signaling pathways activated through axon-glia cellular adhesion molecules (reviewed in [142]). At least one of these adhesion molecules, Neuregulin 1, has been shown to depend on vesicle trafficking for its expression in the proper location on the membrane [108]. One pathway promoting oligodendrocyte proliferation and (re-)myelination is by activation of the Notch-pathway by F3/Contactin [63]. Upregulation of F3/Contactin in denuded axons as observed in MS lesions [109] is considered essential for successful remyelination [117]. F3/Contactin travels to the plasma membrane via a route that bypasses the Golgi apparatus [14, 15]. Although this means that delivery of the protein to the membrane can be facilitated in a microtubule-independent manner, the polarized trafficking of membranes to specific compartments still requires delivery through endosomes via the cytoskeleton [74, 85]. A less efficient transport would lead to a slower initiation of remyelination, prolonging the period of demyelination stress and thereby increasing the risk of axonal loss.

## Local inflammation and neurodegeneration aggravates transport deficits

As described in the previous paragraphs, there are various ways in which axonal transport deficits influence neurodegeneration. However, the opposite is also true, with the biochemical environment existing during inflammation and neurodegeneration affecting the transport system. This is valid for mechanisms seen in a variety of neurodegenerative disorders, such as glutamate toxicity and mitochondrial decay, as well as for events more specific to MS, such as inflammation and demyelination.

### Demyelination leads to dysregulation of axonal transport

Although axonal transport functions properly in individual neurons in culture, several studies have shown that myelination plays an important role in its regulation. In co-cultures of neurons and oligodendrocytes, myelination is often incomplete, with only parts of the axon covered with a myelin sheath. These myelinated sections show a local slowing of axonal transport, resulting in a locally increased axon diameter [105]. Oligodendrocyte-axon interactions lead to specialization of segments of the axons around the nodes of Ranvier. These paranodal regions show a significantly larger mitochondrial content and increased speed of mitochondrial transport. In a myelin deficient mouse, mitochondria are localized throughout the axon and transported with a uniform speed [112]. Furthermore, in mice with a null mutation of the myelin *Plp* gene, a model system for hereditary spastic paraplegia type 2, an impairment of both anterograde and retrograde transport in axons was observed [36]. A recent study of this model showed microtubule pathology, mitochondrial degeneration and reduced ATP in the axon [158]. One could argue that this impairment is due to the energy deficit and calcium influx associated with demyelination. However, a similar phenotype is observed in the CNP knockout mouse, in which myelin assembly is normal but only the signaling between oligodendrocyte and axon is disrupted [84]. One possible explanation for the disruption of transport could be a local drop in ATP, since the mitochondria in the axon partially depend on lactate supplied by oligodendrocytes [86, 125].

### Inflammation leads to cytoskeleton destabilization

In active inflammatory lesions, the activated T-cells that have infiltrated the CNS induce microglia to produce tumor necrosis factor alpha (TNF-α, [23]). Apart from its function in regulating the immune response, exposing cells to high concentrations of this cytokine also leads to destabilization of microtubules and loss of cell integrity [115, 132]. As of yet, the exact mechanism through which TNF-α leads to microtubule destabilization remains unknown. It is possible this effect is mediated through glutamate toxicity, as TNF-α induces secretion of glutamate while at the same time decreasing the glutamate uptake by glia cells [113]. However, TNF-α also leads directly to dissociation of KIF5B from the microtubule through phosphorylation of c-Jun N-terminal Kinase [137].

Activated microglia not only produces TNF-α and other cytokines, but also expresses the enzyme inducible nitric oxide synthase (iNOS, [30]. Nitric oxide (NO) acts as an almost universal signaling molecule, affecting a large variety of molecular pathways. This makes it hard to isolate its effect on the cytoskeleton. An extensive body of research exists on the action of NO on the plant cytoskeleton. In plants, stimulation of cells with NO leads to depolymerization of microtubules [131], leading to an overall disorganization of both the actin [76] and the microtubule network [92]. Although less thoroughly studied, the same mechanism is also present in mammalian neurons. In these cells, stimulation with NO leads to reconfiguration of the microtubule network through nitrosylation of MAP1B, resulting in growth cone collapse and axon retraction [139]. This shows that the chemical environment associated with neuroinflammation is in itself already capable of disrupting microtubule-associated axonal transport.

### Mitochondrial decay inhibits axonal transport

As mentioned previously, changes in mitochondria density, mobility and activity are a common finding in MS neuropathology [101, 153]. Although these changes initially are aimed at protection of the axon by providing a steady supply of ATP, if these mitochondria are not replaced they become a prime contributor to neurodegeneration [75]. One of the mechanisms through which decaying mitochondria lead to axonal loss is by disturbing the calcium homeostasis [120]. As with glutamate excitotoxicity, the resulting increase in intracellular calcium will lead to a cascade of events, including transport disruption, finally resulting in apoptosis or necrosis. Defects in the respiratory complexes can lead to excess production of radical oxygen species (ROS). The oxidative stress caused by the reaction of these free radicals with proteins in the neuron contributes to neurodegeneration [90, 146]. One of the first effects of artificially induced oxidative stress is inhibition of axonal transport, occurring hours before any effect is seen on other cellular structures [39]. The same study showed that depletion of ATP, another consequence of mitochondrial degradation, will also inhibit axonal transport of mitochondria and Golgi-derived vesicles. As mitochondria fail, axonal transport will become dysfunctional as well.

### Effects of glutamate toxicity on the cytoskeleton

Glutamate excitotoxicity has long been recognized as a contributor to neurodegeneration in a variety of neurological disorders [72], including MS [45, 81, 116]. Under normal circumstances, glutamate can bind to channels in the plasma membrane, generating a small and strictly controlled flow of ions. When a neuron is overstimulated with glutamate, the size of this ion flux is increased, resulting in a rising calcium concentration in the cytoplasm. This accumulation in turn triggers several signaling cascades, finally resulting in apoptosis (reviewed in [145]). Before the levels initiating apoptosis are reached, this increase in intracellular calcium already has a detrimental effect on the cytoskeleton. The infrastructure for transport is degraded, as both microtubules [102] and intermediate filaments [26] are destabilized and lost. Since the organization of the microtubule skeleton is essential for axon structure and integrity [78], this degradation will eventually lead to neurodegeneration. In cultured neurons, the influx of calcium caused by glutamate is indeed sufficient to slow down or inhibit fast axonal transport [2, 60]. This could explain why an estimated 50% of demyelinated axons in the brain of MS patients show fragmentation of the neurofilament network and reduced organelle content [33].

Apart from the direct effect of glutamate toxicity on microtubule stability, there is also an indirect effect on transport through alteration of posttranslational modifications (PMTs). These modifications influence microtubule dynamics, but also function as traffic rules regulating binding affinity of molecules including motor proteins [65, 67, 135]. A combination of at least two of these modifications, acetylation and detyrosination, enhances the binding of Kinesin-1 (Kif5) to microtubules and its motor activity as well as its preferential localization to the axon [53, 77, 121]. A decrease in the level of acetylated α-tubulin in a mouse model induced severe axonal transport deficits. Clinically this resulted in neurological deficits resembling either Charcot-Marie-Tooth disease or distal hereditary motor neuropathy, depending on the exact mutation generated in the deacetylase enzyme HDAC6. Treating the animals with an inhibitor of HDAC6 rescued the transport deficits and led to disappearance of the clinical phenotype [35]. Microtubule modifications are not static, but can vary over time. For example, neuronal activity leads to a local increase of microtubule polyglutamylation causing a reduction of Kif5 mobility and cargo delivering [97] as well as an increase in acetylation of α-tubulin [114]. In contrast, loss of polyglutamylation leads to abnormal targeting of Kif1A and a decrease in density of synaptic vesicles [64]. Decreases in acetylation have also been suggested to play a role in several human neurodegenerative disorders, most notably Alzheimer’s disease [57, 160]. If activation of glutamate receptors leads to changes in tubulin acetylation [114], it is very likely that glutamate excitotoxity will also affect the balance between the different PMTs, and therefore, disrupt the proper regulation of axonal transport. Further research is required to determine if such a mechanism plays a role in neurodegeneration in MS.

## The downward spiral

**Fig. 1:**
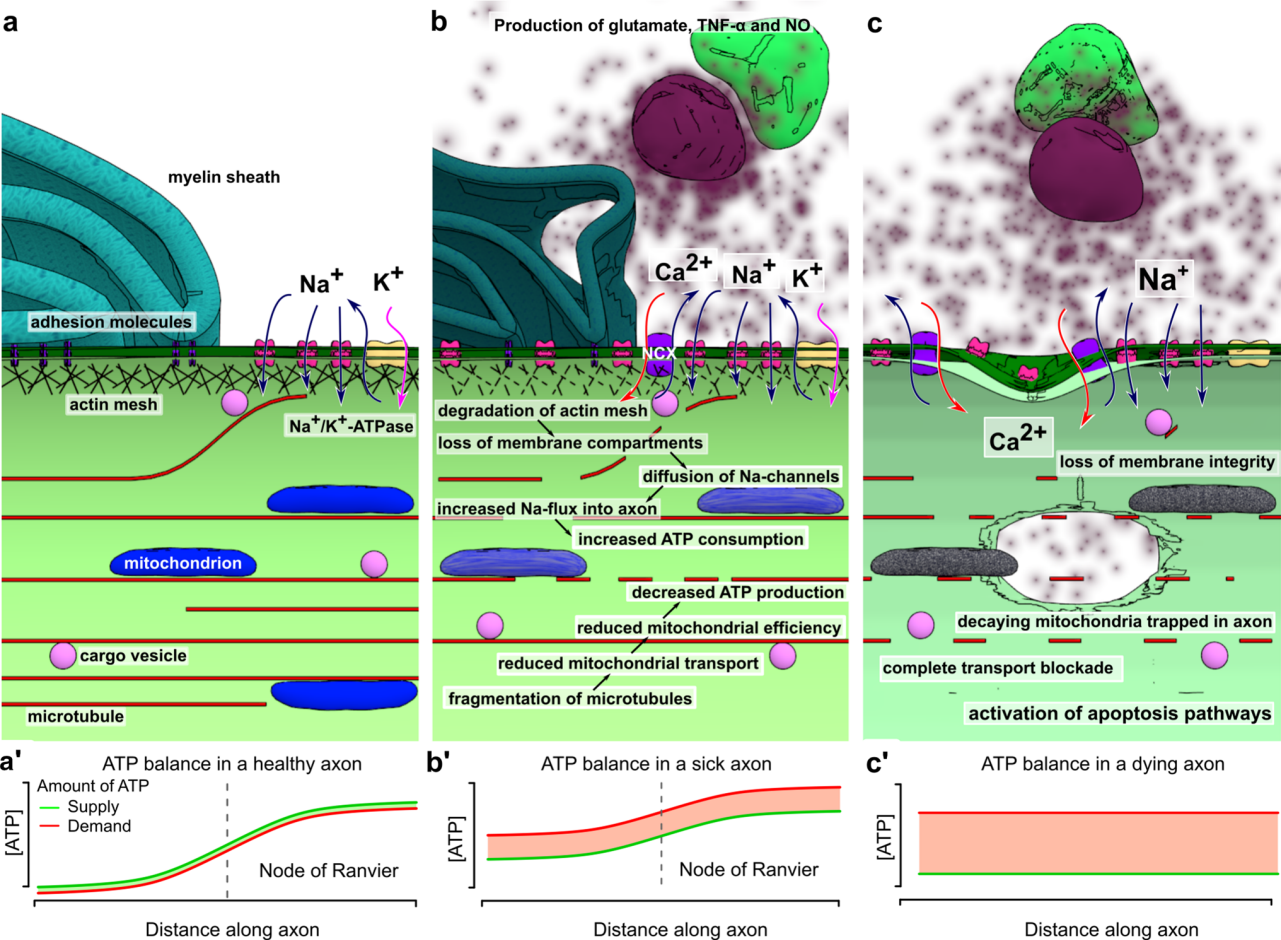
Transport defects and axonal degeneration. In the healthy axon (**a**), the membrane is organized in different compartments through interaction of adhesion molecules and other membrane proteins with the underlying actin mesh. In the nodes of Ranvier, this interaction prevents the ion channels from diffusing out of the nodes. The ion flow through these channels has to be compensated by a similar magnitude outflow to restore membrane potential. The main protein complex involved in this outflow is Na^+^/K^+^-ATPase, an enzyme that exchanges intracellular sodium for extracellular potassium, hydrolyzing an ATP molecule in the process. This mechanism results in a peak in ATP demand at the nodes of Ranvier compared to the rest of the axon. The axonal transport system matches this demand by guiding a constant flow of mitochondria into the nodes and anchoring them to the microtubules where demand is highest, resulting in a careful balance between supply and demand (**a’**). In the micro-environment created by neuroinflammation this balance is disturbed (**a**). Microglia, activated by T-lymphocytes infiltrating the CNS, produce a mixture of different compounds such as glutamate, TNF-α and nitric oxide, causing degradation of the actin network and fragmentation of the microtubule cytoskeleton. As a result, the membrane compartments fall apart and ion channels start to diffuse along the membrane. In addition, the reduced mitochondrial transport along the fragmented microtubules results in aging mitochondria being stuck in the axon, leading to decreased mitochondrial efficiency. The diffusion of sodium channels and their activation through increased glutamate levels will lead to a peak in ATP consumption through Na^+^/K^+^-ATPase, which is no longer restricted to the nodes. As mitochondria can no longer be freely redistributed, the transport system is not able to match ATP supply to demand (**b’**). When the ATP levels drop significantly, a backup mechanism enables the neuron to maintain its membrane potential by exchanging intracellular sodium for extracellular calcium. At this point, the damage to the axon is still reversible. If the inflammatory environment disappears and the transport defects are corrected, the situation in (**a**) can be restored. If intracellular calcium levels keep rising, damage to the transport system accumulates and becomes irreversible (**c**). The continued exposure to high levels of glutamate completely disintegrates the actin mesh. Transport along microtubules ceases as they are depolymerized and motor proteins are unable to function because of low ATP levels caused by mitochondrial dysfunction (**c’**). The increase in intracellular calcium activates a variety of enzymes, leading to loss of membrane integrity and finally loss axonal degradation

From the studies reviewed in this article, a picture emerges of axonal transport deficits as both cause and consequence of neuronal degeneration. In the healthy axon, fast intracellular transport is supported by a dense network of microtubules. Molecular motors transport a variety of cargoes using this infrastructure, including mitochondria and vesicles containing cellular adhesion molecules, amongst many others. This continuous stream of supplies is essential in meeting the energy demand of the axon through local ATP production, as well as maintaining contact with oligodendrocytes through cell–cell adhesions. The membrane is divided into several compartments, maintained by interaction of membrane proteins with the (actin) cytoskeleton. These domains prohibit diffusion of glutamate receptors outside of the nodes of Ranvier, concentrating the peak demand for ATP to these areas of the axon. Through post-translational modifications of microtubules and local concentration of anchoring proteins, a large number of mitochondria are retained in these nodes, producing ATP where it is most needed (Fig. 1a).

Failure of axonal transport has severe consequences for the axon. Local peaks in energy demand can no longer be answered by increased transport of mitochondria. Although mitochondria are able to divide and fuse in the axon [5], defective mitochondria are usually transported back to the perinuclear region for mitophagy [151]. As transport fails, these mitochondria will remain in the axon and become a source of radical oxygen species (ROS) and calcium. Since both anterograde and retrograde transport is affected, proteins will start to accumulate in the axon. When the organization of the cytoskeleton further deteriorates and transport of adhesion molecules to the membrane slows down, the boundaries between the nodes of Ranvier, the paranodal regions and the remainder of the membrane will become less clearly demarcated. This weakens the connections between the axon and its myelin sheath and allows for ion channels to diffuse outside of the nodes of Ranvier. The demand for ATP rises and becomes more uniformly spread throughout the axon, instead of being concentrated in the nodes of Ranvier. At the same time, the ROS released by mitochondria, as well as the dropping levels of ATP further decrease the efficiency of the axonal transport system (Fig. 1b).

In the context of MS, the molecular environment caused by neuroinflammation is an additional detrimental factor to axonal transport. T-cells infiltrating the central nervous system induce the activation of microglia. Microglia in turn becomes a source of a cocktail of chemicals, including TNF-α and NO. High concentrations of NO lead to depolymerization of the microtubule network, further hampering transport. TNF-α disrupts glutamate homeostasis, simultaneously stimulating its release and inhibiting re-uptake. The axon is now exposed to high levels of glutamate, which are even more harmful due to the increased and more diffuse concentration of sodium channels in the axon membrane. The resulting influx of sodium has to be compensated through action of the Na^+^/K^+^-ATPase, one of the most energy-consuming processes in the cell [62].

At this point, the neuron is reaching a critical limit. If the toxic effects of neuroinflammation disappear in time and the damage to the transport system is limited, the pathways leading to neurodegeneration could be reversed. Microtubules will grow back into the axon, transport will resume and the damage can be repaired. As membrane domains are reformed, even remyelination becomes possible. However, for this to happen, all conditions must be exactly right. If for example genetic variations in one of the motor proteins lead to a less efficient transport capacity, the balance might tip towards neurodegeneration. If the remaining ATP supplies are depleted, the intracellular sodium concentration raises to critical levels. This leads to a reversal of the calcium current along the Na^+^/Ca^2+^ exchanger, allowing calcium to flow into the neuron while pumping sodium out, allowing the neuron to maintain its action potential [162]. If this situation persists for too long, the intracellular calcium concentration will be high enough to activate a number of enzyme systems, finally resulting in loss of membrane integrity and apoptosis (Fig. 1c).

**Fig. 2:**
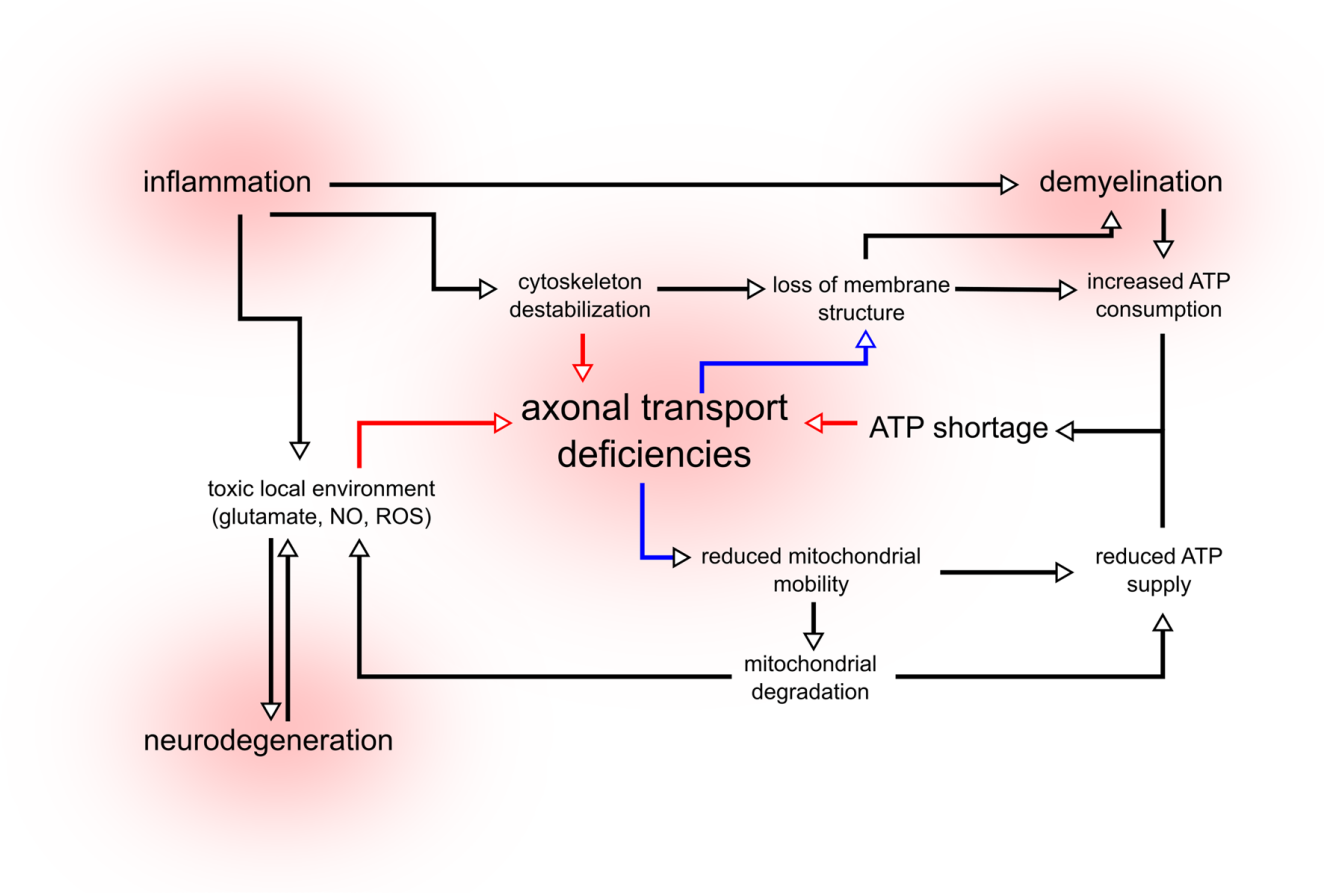
The cycle of neurodegeneration. In MS, both inflammation and neurodegeneration lead to a toxic local environment composed of high concentrations of glutamate, nitric oxide (NO) and radical oxygen species (ROS). These chemicals destabilize the cytoskeleton and affect the function of the axonal transport system. Inflammation also leads to demyelination, exposing large sections of the axon to the hostile micro-environment and increasing the demand for ATP. As transport efficiency is decreased, transport of mitochondria is impaired, leading to a reduced supply of ATP and accumulation of degrading mitochondria in the axon. These mitochondria become an additional source of ROS, contributing to toxicity. Due to transport failure, the constant flow of membrane lipids and proteins diminishes, leading to the loss of membrane structure and integrity. This further contributes to demyelination and prevents remyelination. The increased demand for ATP combined with the reduced supply leads to ATP shortages, preventing motor protein function. Through this loop, axonal transport deficiencies, mitochondrial defects and inflammation amplify each other, creating a positive feedback system that leads to neurodegeneration

Since the first MS cases were neuropathologically examined, several theories have been proposed on the role of neurodegeneration. MS has been considered a purely autoimmune disorder, but also as a primary degenerative disorder with a secondary immune response [27, 140]. Even in brain material from MS patients, some individuals show demyelinated lesions with primarily T cell mediated inflammation, while others show only oligodendrocyte dystrophy [95]. The model in Fig. 2 attempts to reconcile these quite distinct hypotheses. We suggest that these two possible etiological pathways eventually may lead to the same vicious circle towards neurodegeneration. Both inflammation and degeneration can trigger axonal transport deficiencies, resulting in a reduced transport of mitochondria and finally ATP shortages. This model could partially explain the clinical variability observed in MS patients. If a patient has a genetic background that results in reduced transport effectiveness, there is an increased risk of developing the primary progressive form of MS (PPMS). This is consisting with the finding that in PPMS, involvement of the corticospinal tracts is more pronounced [1], as the relatively long axons forming these tracts are highly depending on axonal transport for survival. However, the same patient with a transport system working at full efficiency would be more likely to develop relapsing-remitting MS (RRMS), characterized by high inflammatory activity but relatively little neurodegeneration [143]. Based on this model, we would predict that a correlation exists between transport efficiency and the clinical delay between diagnosis and secondary progression.

In the pathogenesis of MS, deficits in axonal transport can contribute to neuropathology, but the reverse is also true. The effects of demyelination and inflammation on the microtubule cytoskeleton initiate and amplify a chain of events resulting in axonal loss (summarized in Fig. 2). Interrupting the spiral of neurodegeneration is the only way to prevent the clinical progression seen in MS patients. We propose that a variety of therapeutic approaches could prove equally effective. Microtubule stabilizing drugs could prevent the loss of axonal infrastructure, while medication targeting mitochondria preserves the local production of ATP and prevents the leakage of calcium and ROS. Anti-inflammatory drugs diminish the neuronal stress caused by exposure to TNF-α, NO and glutamate, while NMDA-receptor blockers and calcium chelators reduce the toxic effects of glutamate excitotoxicity. As of yet, there are no drugs present that can increase the efficiency of the axonal transport system, although substances such as tubastatin that modify post-translational modification are interesting candidates [35]. The damage that has already occurred cannot be undone, but these approaches can increase the probability that an axon will survive the toxic environment of an active MS lesion.

### Concluding remarks

The association between axonal transport and neurodegeneration is complex and bidirectional. Deficiencies in intracellular transport can lead to a positive feedback loop, a loop in which reduced transport of mitochondria and other components leads to local ATP shortages, which further hamper transport (Fig. 2). If the circumstances interfering with transport persist for a certain amount of time, this loop will inevitably lead to axonal loss. There is substantial evidence that such a loop could play a role in a large variety of neurodegenerative disorders (reviewed in [59, 103]). In MS, this cycle becomes even more vicious because of the contribution of inflammation and demyelination, further increasing both transport defects and ATP demand. This feedback loop would explain a number of puzzling findings in MS, since it suggests that a number of different initial events will lead to the same outcome. A combination of deficiencies in mitochondrial activity, a hampered axonal transport system or a specific immune makeup will all lead to the same outcome, but in each patient the emphasis will be different. Some MS patients with a severe mitochondrial or transport phenotype will show a large amount of neurodegeneration with little inflammation, while other patients will be more on the inflammatory end of this spectrum. Such a spectrum of disease could explain the large variability in disease progression and therapy response observed in MS patients. This neurodegenerative loop also offers new hypothetical approaches towards MS treatment, since it suggests that weakening any part of the loop could reduce neurodegeneration, and therefore, disease progression. Therapy focused on restoring the ATP balance or increasing transport efficiency would weaken the loop and slow down neurodegeneration. Monitoring the effect of a therapy targeting axonal transport is a challenge in itself. In animals, transport can be observed directly using in vivo microscopy of the sciatic nerve [47], a technique not feasible in humans. However, in the near future it might become possible to measure transport in the neurons of the retina [124]. Developing these neuroprotective strategies and combining it with the immunomodulatory drugs already available to MS patients will, hopefully in the near future, greatly reduce the suffering caused by this debilitating disease.
